# Triggering daily online adaptive radiotherapy in the pelvis: Dosimetric effects and procedural implications of trigger parameter‐value selection

**DOI:** 10.1002/acm2.14060

**Published:** 2023-06-05

**Authors:** Adam D. Yock, Mahmoud Ahmed, Sarah Masick, Manuel Morales‐Paliza, Christien Kluwe, Ashwin Shinde, Austin Kirschner, Eric Shinohara

**Affiliations:** ^1^ Department of Radiation Oncology Vanderbilt University Medical Center Nashville Tennessee USA

**Keywords:** adaptive radiotherapy, online ART

## Abstract

**Background:**

Online adaptive radiotherapy (ART) can address dosimetric consequences of variations in anatomy by creating a new plan during treatment. However, ART is time‐ and labor‐intensive and should be implemented in a resource‐conscious way. Adaptive triggers composed of parameter‐value pairs may direct the judicious use of online ART.

**Purpose:**

This work analyzed our clinical experience using CBCT‐based daily online ART to demonstrate how a conceptual framework based on adaptive triggers affects the dosimetric and procedural impact of ART.

**Methods:**

Sixteen patients across several pelvic sites were treated with CBCT‐based daily online ART. Differences in standardized dose metrics were compared between the original plan, the original plan recalculated on the daily anatomy, and an adaptive plan. For each metric, trigger values were analyzed in terms of the proportion of treatments adapted and the distribution of metric values.

**Results:**

Target coverage metrics were compromised due to anatomic variation with the average change per treatment ranging from ‐0.90 to ‐0.05 Gy, ‐0.47 to ‐0.02 Gy, ‐0.31 to ‐0.01 Gy, and ‐12.45% to ‐2.65% for PTV D99%, PTV D95%, CTV D99%, and CTV V100%, respectively. These were improved using the adaptive plan (‐0.03 to 0.01 Gy, ‐0.02 to 0.00 Gy, ‐0.03 to 0.00 Gy, and ‐4.70% to 0.00%, respectively). Increasingly strict triggers resulted in a non‐linear increase in the proportion of treatments adapted and improved the distribution of metric values with diminishing returns. Some organ‐at‐risk (OAR) metrics were compromised by anatomic variation and improved using the adaptive plan, but changes in most OAR metrics were randomly distributed.

**Conclusions:**

Daily online ART improved target coverage across multiple pelvic treatment sites and techniques. These effects were larger than those for OAR metrics, suggesting that maintaining target coverage was our primary benefit of CBCT‐based daily online ART. Analyses like these can determine online ART triggers from a cost‐benefit perspective.

## 1 INTRODUCTION

Adaptive radiotherapy (ART)—whereby treatment plans are recreated to account for anatomic change, disease progression, or treatment response—has been shown to improve the dose delivered to patients in the presence of anatomic variations.^1^, ^2^, ^3^, ^4^, ^5^, ^6^, ^7^, ^8^, ^9^, ^10^, ^11^, ^12^, ^13^, ^14^, ^15^, ^16^, ^17^, ^18^ However, adaptive replanning is an involved process that includes repeated structure delineation, plan optimization, and dose calculation.^19^ This introduces additional demands on clinical resources and personnel.^20^ ART must therefore be implemented in a way that maximizes the benefit to the patient without compromising clinical resources or operations.

When implementing an ART program, one must consider the characteristics of the type of variation it is intended to address. What is appropriate for one treatment site or technique may not be appropriate for another. For example, nearly two decades of work have described how head‐and‐neck cancer patients benefit from offline ART workflows that incorporate a small number of replanning efforts spaced throughout the course of treatment.^1^, ^2^, ^3^, ^4^, ^5^, ^6^, ^7^, ^8^ Anatomic changes observed in these patients (e.g., weight loss, tumor shrinkage, and medial drift) tend to trend across multiple treatments.^1^, ^21^, ^22^, ^23^ Therefore, offline ART is an effective technique as it establishes a new baseline designed to track with the changing anatomy. In this context, questions regarding the implementation of ART are focused on the frequency and timing of adaptive interventions.

For an online ART program intended to address large, random, interfractional variations like those observed in the pelvis, the appropriate implementation should take a different form. In the pelvis, the motion and deformation of radiotherapy targets and organs‐at‐risk are largely governed by variable bladder and rectal filling.^24^, ^25^, ^26^, ^27^, ^28^, ^29^, ^30^, ^31^, ^32^, ^33^, ^34^ In this context, an implementation of ART driven by a predetermined frequency and timing of adaptive interventions is likely to be less effective, as the anatomic changes do not follow the patterns that made such a method appropriate for head‐and‐neck cancer patients. Instead, to address anatomic variations like these, adaptive interventions should be conducted *as needed* (i.e., ad hoc). Identifying what constitutes *as needed* in ART is challenging but can be approached through establishing clinical conditions that trigger the adaptive intervention. These *triggers* are circumstances specified by one or more *parameter‐value* pairs that identify when ART is appropriate. The trigger's *parameter* is the particular signal that is monitored, and the trigger's *value* pertains to the specific measurements of that parameter that correspond to the decision of whether or not to adapt.

For example, in many online ART workflows, the original plan can be recalculated on the daily anatomy, and the resulting dose distribution can be compared to the original one or to that of a daily adaptive plan reoptimized on the daily anatomy. Even without any adaptive replanning effort, the difference in any particular dose metric between the original dose distribution and that of the original plan recalculated on the daily anatomy could serve as the ART trigger parameter. This trigger would respond to scenarios where the anatomic change has compromised the dose distribution of the original plan, thereby warranting adaptation. Alternatively, the trigger parameter could be the difference in the same dose metric as above, but as observed between the dose distribution of the original plan recalculated on the daily anatomy and that of the adaptive plan. This trigger would respond to the potential dosimetric improvement of the adaptive plan, regardless of the adequacy of the original plan. This latter scenario does require the completion of adaptive replanning effort or at least requires an estimation of the effect of the adaptive plan. While the adapted plan will likely be dosimetrically superior, even if only by a marginal amount, finalizing and delivering that plan may still require additional tasks or extra personnel that clinics may wish to avoid unless determined to be dosimetrically justified. While some tasks, such as the initial contouring or potential dose calculation of the original plan, may be required regardless of which plan is selected, others, such as comprehensive plan review and quality assurance, may be required only if the delivery of the new adapted plan is likely. The sequence and specific effort required is dependent on the precise implementation of the ART workflow which can vary between vendors and may be subject to change. Regarding the two scenarios above, with triggers based on a compromised dose distribution or the potential of an improved one, represent two different adaptive philosophies. In addition, each can be implemented according to any particular dose metric. Furthermore, for any dose metric, the action limit that signals the need for adaptation can take any value. The large number of possible parameters and values, along with the fact that multiple triggers may be used in combination, generates countless ways to implement online ART.

For this reason, identifying appropriate triggers is critical for the optimal implementation of online ART, particularly for pelvic disease sites that are best adapted *as needed*. Although triggers for patient selection and timing of offline ART have been investigated,^9^, ^10^, ^11^, ^12^, ^13^, ^14^, ^15^, ^16^, ^17^ the optimal triggers to determine the need for online ART on any given day remain unknown. Not only is the number of possible triggers very large, but clinical implementation of online ART as facilitated by new technologies is relatively recent,^18^, ^20^, ^35^, ^36^, ^37^, ^38^, ^39^, ^40^, ^41^, ^42^, ^43^ and the clinical experience required to determine the effect of any particular trigger remains limited.

The purpose of this work was to analyze our clinical experience using CBCT‐based daily online ART in order to demonstrate a conceptual framework of how adaptive triggers may be used to determine the need for adaptation on a daily basis, as well as to present the varied dosimetric and procedural implications observed using this framework.

## 2 METHODS

### 2.1 Patients

Patients treated at our institution in the last 27 months using CBCT‐based daily online ART (Ethos Therapy, Varian Medical Systems, Palo Alto, California, USA) were considered for this study. Disease sites were all located within the pelvis and included cancers of the prostate and bladder. Treatment sites and techniques that were highly standardized with respect to our institutional online ART implementation and that had at least two patients successfully complete therapy were included for analysis. This included 16 patients comprising 320 total treatment fractions distributed across (1) hypofractionated treatment to the bladder (55 Gy in 20 days, 2.75 Gy/day, four patients representing 80 treatment factions), (2) treatment of a sequential boost to the post‐operative prostate fossa (19.8 Gy in 11 days, 1.8 Gy/day, two patients representing 22 treatment factions), (3) hypofractionated treatment to the prostate plus proximal seminal vesicles (70.2 Gy in 26 days, 2.7 Gy/day, 8 patients representing 208 treatment factions), and (4) prostate stereotactic body radiotherapy treatment (40 Gy in 5 days, 8 Gy/day, two patients representing 10 treatment factions). CTV to PTV margin expansions for these patients were as follows: bladder—3–5 mm in all directions; prostate fossa—3 mm posteriorly and 3–5 mm in all other directions; prostate plus proximal seminal vesicles—3 mm posteriorly and 3–5 mm in all other directions; and prostate SBRT—3 mm superiorly and posteriorly, and 4 mm to the left, right, inferior, and anterior.

The Ethos online ART workflow has been described in detail elsewhere.^20^, ^42^, ^43^ In short, following the acquisition of a daily CBCT, the system uses deformable image registration and AI‐based auto‐contouring tools to delineate influential normal tissues followed by treatment targets, as well as to generate a synthetic CT. Subsequently, the system recalculates the dose of the original plan on the synthetic CT and re‐optimizes a new adaptive plan. The vast majority of all structure delineation and plan evaluation tasks during both the initial treatment planning and each daily treatment were overseen by either one of two individual physicians (CK, AS).

### 2.2 Standardized dose metrics

For each patient, values of a standardized set of dose metrics were collected from the following three dose distributions available for each treatment:

- The dose distribution of the “original plan” calculated on the “simulation CT anatomy” (i.e., the *Reference Dose*), which did not vary between treatments,
- The dose distribution of the “original plan” recalculated on the “daily anatomy” (i.e., the *Scheduled Dose*),
- The dose distribution of the “adaptive plan” reoptimized on the “daily anatomy” (i.e., the *Adapted Dose*).

The complete set of metrics is listed in Table 1. These metrics were not necessarily the objectives used during plan optimization but were considered adequate to characterize the dose delivered to the targets and organs‐at‐risk across different patients, treatment sites, and treatment techniques. Instances where metrics did not correspond to reasonable objectives or did not reflect sufficient dose for comparison due to the particular target or prescription dose were noted. For each metric, the difference in the value between the Scheduled Dose and the Reference Dose (SCH‐REF) was determined, as was the difference in the value between the Adapted Dose and the Reference Dose (ADP‐REF). These differences reflected the degree to which the Scheduled and Adapted Doses matched the Reference Dose which was reviewed and approved prior to treatment and which represented the anticipated value of each metric. The dose value of each metric was determined from the cumulative dose‐volume histogram as presented in the treatment planning system (Ethos Therapy; Varian Medical Systems, Palo Alto, California, USA), and the relative values of the SCH‐REF and ADP‐REF were calculated and summarized with descriptive statistics.

**TABLE 1:** Standardized dose metrics.

Structure	Dose metrics
Planning target volume	D99%, D95%, and D0.03cc
Clinical target volume	D99%, V100%, and D0.03cc
Bowel	D2cc, V55Gy^a^, and D0.03cc
Bladder	V65Gy^a^, V40Gy^a^, and D0.03cc
Rectum	V65Gy^a^, V40Gy^a^, and D0.03cc
Left femur head & neck	D0.03cc
Right femur head & neck	D0.03cc

Dx%, Dose to x% of structure (Gy); Dxcc, Dose to x cubic centimeters of structure (Gy); Vx%, Volume of structure receiving at least x% of prescription dose (%); VxGy, Volume of structure receiving at least x Gy (%).

^a^ Metric scaled by number of treatments to determine corresponding dose limit per treatment.

### 2.3 Trigger parameter‐value pairs

The SCH‐REF and ADP‐REF differences in each metric were considered potential adaptive trigger parameters, and a set of integer values spanning the range of observed differences were analyzed as possible trigger value action levels. The proportion of treatments where ART was triggered was calculated for each of these parameter‐value pairs. In addition, the distribution of values of each metric that would have resulted from a parameter‐value pair acting as a trigger was determined by combining the values from Adapted Doses for treatments where ART was triggered with those from Scheduled Doses for treatments where it was not.

## 3 RESULTS

### 3.1 Standardized dose metric difference distributions

**FIGURE 1:**
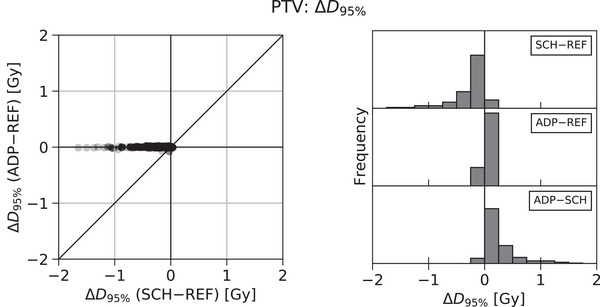
Differences in PTV D95% according to Reference, Scheduled, and Adapted Dose distributions for patients treated to the prostate plus proximal seminal vesicles.

Figure 1a presents a scatterplot showing the difference in value between the Scheduled Dose and the Reference Dose (SCH‐REF) and between the Adapted Dose and the Reference Dose (ADP‐REF) for an example target coverage metric, the PTV D95% for patients treated to the prostate plus proximal seminal vesicles. As the x‐axis measures the SCH‐REF difference, positive and negative values represent when the Scheduled Dose value was greater than or less than the Reference Dose value, respectively. Similarly, the y‐axis (ADP‐REF) discriminates whether the Adapted Dose value was greater than or less than the Reference Dose value. The direct comparison of Adapted Dose and Scheduled Dose values (ADP‐SCH) can be observed from the 45° line corresponding to y = x. Figure 1b presents the corresponding histograms of the SCH‐REF, ADP‐REF, and ADP‐SCH differences. Together, Figure 1a and 1b demonstrate how the Scheduled and Adapted Dose values compare to that of the original Reference Dose.

For patients treated to the prostate plus proximal seminal vesicles, the distribution of SCH‐REF differences in PTV D95% was broad and biased toward negative values (representing compromised target coverage). The differences had a median value of ‐0.14 Gy and an interquartile range (IQR) of 0.28 Gy. Meanwhile, the distribution of ADP‐REF differences was narrower and more centered around zero (representing greater equivalence to the Reference Dose). Their median value was 0.00 Gy with an IQR of 0.01 Gy. Lastly, the median value of the ADP‐SCH differences was 0.15 Gy with an IQR of 0.29 Gy.

Patterns in the SCH‐REF, ADP‐REF, and ADP‐SCH differences of the PTV D95% metric for patients treated to the prostate plus proximal seminal vesicles were similar to those observed for all target coverage metrics (PTV: D99% and D95%, CTV: D99% and V100%) across all four treatment sites and techniques, although the magnitude of the changes did vary. However, considerable interpatient variation was also observed, even within a particular treatment site and technique.

**FIGURE 2:**
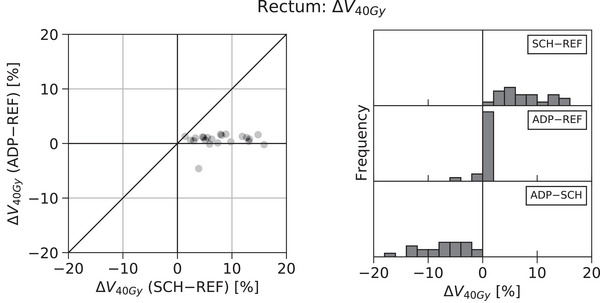
Differences in rectum V40Gy according to Reference, Scheduled, and Adapted Dose distributions for patients treated with a sequential boost to the postoperative prostate fossa.

Figure 2 presents the distributions of SCH‐REF, ADP‐REF, and ADP‐SCH differences for an example organ‐at‐risk (OAR) metric, the V40Gy of the rectum for patients treated with a sequential boost to the post‐operative prostate fossa. As in Figure 1b, the histograms of Figure 2b, show a broad distribution of SCH‐REF differences (median = 6.85%, IQR = 6.73%) and a narrower distribution of ADP‐REF differences more centered around zero (median = 0.90%, IQR = 0.85%). In this case, however, the SCH‐REF distribution is biased toward positive values, representing increased dose to the OAR with the Scheduled Dose, which the ADP‐SCH distribution signifies is decreased with the Adapted Dose (median = ‐6.40%, IQR = 6.45%). Also unlike the PTV D95% metric depicted in Figure 1, which was representative of target coverage metrics across treatment sites and techniques, the example V40Gy metric depicted in Figure 2 did not represent the behavior of other OAR metrics, which largely exhibited more random distributions.

While the PTV D95% and the rectum V40Gy are depicted here as examples, statistical descriptions of the SCH‐REF, ADP‐REF, and ADP‐SCH difference distributions for all metrics are presented in Table 2, each representing a possible adaptive trigger parameter.

**TABLE 2:** Descriptive statistics of changes in standardized dose metric values according to Reference, Scheduled, and Adapted Dose distributions.

		Hypofractionated bladder								Postoperative prostate fossa boost					
		SCH ‐ REF		ADP ‐ REF		ADP ‐ SCH				SCH ‐ REF		ADP ‐ REF		ADP ‐ SCH	
Structure	Metric	Median	IQR	Median	IQR	Median	IQR	Structure	Metric	Median	IQR	Median	IQR	Median	IQR
PTV								PTV							
	D99% (Gy)	−0.87	0.74	−0.01	0.02	0.86	0.68		D99% (Gy)	−0.05	0.15	0.01	0.03	0.05	0.13
	D95% (Gy)	−0.42	0.59	0.00	0.02	0.42	0.60		D95% (Gy)	−0.02	0.04	0.00	0.01	0.02	0.04
	D0.03cc (Gy)	0.03	0.13	−0.01	0.02	−0.04	0.15		D0.03cc (Gy)	0.02	0.02	0.02	0.04	0.00	0.04
CTV								CTV							
	D99% (Gy)	−0.31	0.65	0.00	0.01	0.31	0.64		D99% (Gy)	−0.01	0.03	0.00	0.01	0.01	0.03
	V100% (%)	−4.50	14.10	0.00	0.00	4.45	14.73		V100% (%)	−2.65	7.53	−0.05	1.75	2.60	7.55
	D0.03cc (Gy)	0.05	0.06	0.01	0.03	−0.03	0.08		D0.03cc (Gy)	0.01	0.02	−0.01	0.05	0.00	0.06
Bowel								Bowel							
	D2cc (Gy)	0.07	0.27	0.00	0.10	−0.02	0.40		D2cc (Gy)	0.09	0.20	0.05	0.07	−0.02	0.11
	V55Gy (%)	0.30	1.73	0.00	0.33	−0.15	2.50		V55Gy (%)	0.00	0.00	0.00	0.00	0.00	0.00
	D0.03cc (Gy)	0.06	0.12	0.00	0.08	−0.07	0.17		D0.03cc (Gy)	0.44	0.97	0.18	0.16	−0.16	0.54
Bladder								Bladder							
	V65Gy (%)	na	na	na	na	na	na		V65Gy (%)	2.05	9.55	3.25	10.50	0.25	1.48
	V40Gy (%)	na	na	na	na	na	na		V40Gy (%)	3.05	18.50	2.15	16.90	0.10	3.00
	D0.03cc (Gy)	na	na	na	na	na	na		D0.03cc (Gy)	0.00	0.03	0.00	0.04	0.00	0.03
Rectum								Rectum							
	V65Gy (%)	–	–	–	–	–	–		V65Gy (%)	4.00	4.43	−0.45	3.05	−4.30	5.40
	V40Gy (%)	0.00	0.10	0.00	0.30	0.00	0.00		V40Gy (%)	6.85	6.73	0.90	0.85	−6.40	6.45
	D0.03cc (Gy)	0.21	0.35	0.11	0.27	−0.08	0.22		D0.03cc (Gy)	0.03	0.07	−0.01	0.02	−0.03	0.04
L & R Femur								L & R Femur							
	D0.03cc (Gy)	0.00	0.09	0.05	0.23	0.05	0.25		D0.03cc (Gy)	0.01	0.05	0.00	0.14	−0.03	0.13

		Hypofractionated prostate and proximal seminal vesicles								Prostate stereotactic body radiotherapy					
		SCH ‐ REF		ADP ‐ REF		ADP ‐ SCH				SCH ‐ REF		ADP ‐ REF		ADP ‐ SCH	
Structure	Metric	Median	IQR	Median	IQR	Median	IQR	Structure	Metric	Median	IQR	Median	IQR	Median	IQR
PTV								PTV							
	D99% (Gy)	−0.44	0.53	−0.01	0.05	0.43	0.53		D99% (Gy)	−0.90	0.65	−0.03	0.06	0.91	0.59
	D95% (Gy)	−0.14	0.28	0.00	0.01	0.15	0.29		D95% (Gy)	−0.47	0.19	−0.02	0.03	0.45	0.23
	D0.03cc (Gy)	0.00	0.06	0.00	0.05	0.00	0.08		D0.03cc (Gy)	−0.12	0.07	−0.20	0.18	−0.05	0.16
CTV								CTV							
	D99% (Gy)	−0.06	0.18	0.00	0.02	0.06	0.18		D99% (Gy)	−0.25	0.27	−0.03	0.03	0.24	0.31
	V100% (%)	−4.70	20.53	0.00	0.63	4.75	21.88		V100% (%)	−12.45	20.08	−4.70	10.80	5.45	10.20
	D0.03cc (Gy)	0.00	0.05	0.00	0.05	0.00	0.08		D0.03cc (Gy)	−0.14	0.07	−0.15	0.21	−0.01	0.17
Bowel								Bowel							
	D2cc (Gy)	0.07	0.48	0.02	0.54	0.00	0.21		D2cc (Gy)	0.03	0.08	0.04	0.08	0.01	0.04
	V55Gy (%)	0.00	0.10	0.00	0.00	0.00	0.00		V55Gy (%)	–	–	–	–	–	–
	D0.03cc (Gy)	0.41	0.79	0.10	0.99	−0.03	0.37		D0.03cc (Gy)	0.03	0.09	0.03	0.10	0.03	0.04
Bladder								Bladder							
	V65Gy (%)	1.10	4.93	1.95	7.30	0.75	3.63		V65Gy (%)	–	–	–	–	–	–
	V40Gy (%)	4.70	16.28	4.85	21.63	0.90	8.13		V40Gy (%)	0.00	0.10	0.00	0.00	0.00	0.10
	D0.03cc (Gy)	−0.01	0.07	0.00	0.07	0.00	0.08		D0.03cc (Gy)	0.01	0.24	−0.02	0.06	−0.06	0.20
Rectum								Rectum							
	V65Gy (%)	1.50	4.80	1.70	2.53	0.40	3.90		V65Gy (%)	–	–	–	–	–	–
	V40Gy (%)	1.90	10.00	0.80	4.20	−0.50	8.85		V40Gy (%)	0.00	0.08	–	–	0.00	0.08
	D0.03cc (Gy)	0.01	0.06	0.02	0.04	0.00	0.06		D0.03cc (Gy)	0.29	1.09	0.20	0.34	−0.14	0.72
L & R Femur								L & R Femur							
	D0.03cc (Gy)	0.01	0.07	0.09	0.21	0.05	0.20		D0.03cc (Gy)	−0.04	0.07	−0.35	0.85	−0.12	0.61

ADP, Adapted Dose; IQR, Interquartile Range; na, Dose constraints not appropriate given target; ( ‐ ), Inadequate dose for comparison; REF, Reference Dose; SCH, Scheduled Dose.

### 3.2 Effects of trigger parameter‐value pairs

**FIGURE 3:**
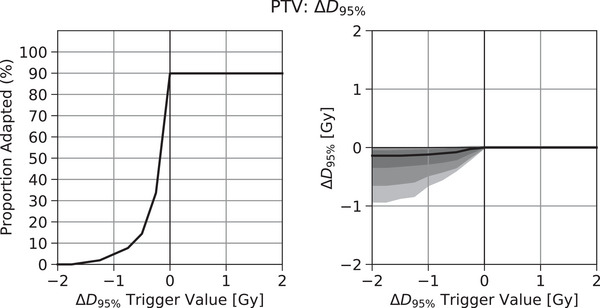
Effect of the SCH‐REF trigger value for PTV D95% (x‐axis) on (a) the proportion of treatments adapted (y‐axis) and on (b) the resulting distribution of changes in PTV D95% values (y‐axis) for patients treated to the prostate plus proximal seminal vesicles. Bands in (b) are bounded by the 5th, 10th, 25th, 50th, 75th, 90th, and 95th percentile values.

Figure 3 depicts the effects that result from considering the SCH‐REF difference as an example adaptive trigger parameter for the PTV D95% for patients treated to the prostate plus proximal seminal vesicles. The x‐axis of both Figure 3a and 3b represents the trigger value for that parameter while the y‐axis depicts the effect of this value on the proportion of treatments adapted (3a) or the resulting distribution of changes in that metric's value (3b). For the PTV D95% metric, as the SCH‐REF trigger value increases, so does the proportion of treatments adapted. Figure 3a shows that the increase in proportion of treatments adapted was non‐linear exhibiting a shallow rate of increase at the lowest trigger values that becomes very steep as SCH‐REF approaches 0%. This non‐linearity characterizes the potential of very unequal effects in the proportion of treatments adapted for equal changes in trigger values. For example, in Figure 3a, changing the trigger value from ‐1.0 to ‐0.5 Gy increases the proportion of treatments adapted from 4.8% to 14.4%. Yet changing the value from ‐0.5 to ‐0.25 Gy (half the previous change) increases the proportion of treatments adapted from 14.4% to 33.7% (twice the previous effect). It is worth noting that the proportion of treatments adapted does not reach 100%. This occurs because a second condition for selecting the adaptive plan is that the Adapted Dose value is preferred over the Scheduled Dose value (e.g., Adapted Dose target coverage > Scheduled Dose target coverage). While this was typically true, it was not always the case for this metric (see Figure 1b, bottom histogram). One possible example scenario where this might occur for a target coverage metric would be when the target shrinks or moves to a higher dose region, increasing target coverage, which the adaptive reoptimization subsequently decreases in a renormalization step.

Figure 3b shows how the distribution of PTV D95% values changes with the trigger value. As increasing the SCH‐REF trigger value increases the proportion of treatments adapted (Figure 3a), the distribution of metric values is made up of an increasing proportion of values from Adapted Doses rather than Scheduled Doses. Because the Adapted Dose values for this metric more closely resemble those of the original Reference Dose (Figure 1), the overall distribution also approaches that of the Reference Dose (Figure 3b). For example, the 10^th^ percentile change in PTV D95% increases from ‐0.65 to ‐0.48 to ‐0.35 Gy for trigger values at ‐2.0, ‐1.0, and ‐0.5 Gy, respectively.

**FIGURE 4:**
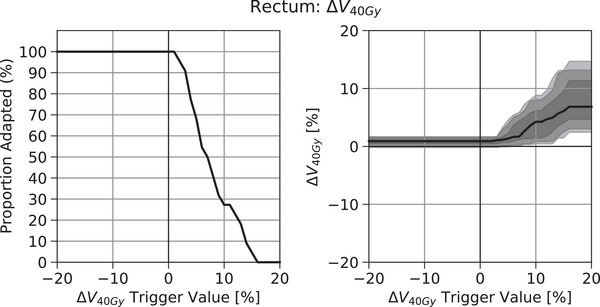
Effect of the SCH‐REF trigger value for rectum V40Gy (x‐axis) on (a) the proportion of treatments adapted (y‐axis) and on (b) the resulting distribution of changes in rectum V40Gy values (y‐axis) for patients treated with a sequential boost to the post‐operative prostate fossa. Bands in (b) are bounded by the 5th, 10th, 25th, 50th, 75th, 90th, and 95th percentile values.

Figure 4 presents the effects of various trigger values for the SCH‐REF parameter for an example OAR metric, the V40Gy of the rectum for patients treated with a sequential boost to the post‐operative prostate fossa. Unlike for target coverage metrics, *decreasing* the SCH‐REF trigger value corresponds to an increasingly strict trigger, which, in turn, increases the proportion of treatments adapted and makes the resulting distribution of metric values more similar to that of the Reference Dose.

The observed increase in the proportion of treatments adapted for each metric as depicted in Figures 3 and 4 are also somewhat characterized by the descriptive statistics of Table 2. The median values listed in Table 2 correspond to the trigger value that results in adapting 50% of treatments, and the IQR denotes the spread of trigger values that encompass the central 50% of observed changes in the metric value.

## 4 DISCUSSION

This work leveraged our clinical experience using CBCT‐based daily online ART to demonstrate a conceptual framework of adaptive trigger selection and to present the varied effects of this framework for multiple pelvic treatment sites and techniques. Our purpose was not to prescribe the use of any specific trigger parameter‐value pair but to demonstrate the concept and key considerations of trigger selection as observed from clinical online ART patients. The dosimetric and procedural effects of adaptive trigger selection depend heavily on the choice of trigger parameter and value. The value of target coverage metrics, which were frequently compromised by changes in patient anatomy, was substantially improved with the reoptimized adaptive plan. This was consistent across the four distinct treatment sites and techniques evaluated, although considerable interpatient effects were also observed. In some instances, OAR metrics were improved with the adaptive plan; however, this varied considerably between metrics. The seemingly random distribution of most OAR metrics suggests that their behavior may be governed by random effects or other unknown factors not explicitly identified here. A nonlinear increase in the number of treatments using the reoptimized adaptive plan occurred when triggers were made increasingly strict. Increasing the proportion of treatments adapted, in turn, improved the distribution of values for metrics that had been compromised by variations in patient anatomy.

The broad improvement in target coverage across treatment sites and techniques can largely be explained by two factors. First, target coverage metrics such as D99%, D95%, and V100% are sensitive to anatomic variations. In an appropriately optimized intensity‐modulated plan, the prescription dose conforms tightly around the target, and the DVH curve exhibits high dose coverage that is followed by a sharp shoulder and steep fall‐off. Little change in anatomy is therefore required for the target to move relative to the region of high dose causing large changes in values of these metrics. Second, target coverage metrics are heavily prioritized during plan optimization. This occurs explicitly with the large weight given to related optimization objectives and with dose renormalization. It also occurs implicitly as the importance of target coverage solicits particular attention from clinicians during plan review and from the fact that the target coverage metric values are likely to be close to decision making thresholds. These factors help explain why target coverage was initially compromised by anatomic variation but readily recovered by the adaptive plan.

The behavior of OAR metrics was less consistent. For some treatment sites and techniques, the adaptive plan did improve dosimetry (e.g., bowel and rectum metrics for patients treated with a sequential boost to the postoperative prostate fossa). However, many others exhibited largely random behavior. This may in part be due to the following considerations. First, many OAR metrics are prioritized lower than target coverage and will therefore be compromised during optimization if they conflict with such goals that have higher priority. Some OAR metrics that are often high priority (e.g., maximum dose) are in very close proximity to the target, making it difficult to optimize a plan that spares this portion of the OAR. In addition, values of OAR metrics are not necessarily near decision making thresholds depending on the prescription and treatment technique. Therefore, OAR metrics may generally not be as sensitive to small anatomic variations as target coverage, and they will not likely be substantially improved by the adaptive plan if their priority is low, if their improvement is costly, or if they are not near a dose limit. If strong forces do not affect the compromise and recovery of OAR metric values, the changes are likely to exhibit largely random behavior.

Ultimately, of course, it is the clinical consequence of these dosimetric changes that are the true assessment of the potential of ART. This data remains forthcoming. Online ART systems such as MR‐linacs and, in particular, CBCT‐guided systems remain new technologies, and clinical trials are currently ongoing to determine their impact across a wide variety of treatment sites. It is worth noting, however, that conventional clinical outcomes are most closely associated with the dosimetry represented by the Scheduled Doses as these doses reflect those of an original plan delivered onto the daily changing anatomy. The difference of these values relative to the Reference Doses largely reflects our previous inability to observe the dose delivered to the patient on a daily basis. Our work presented here was not intended or designed to correlate differences in dose metric values with clinical outcomes, but rather to observe and present the variable behavior of trigger‐value selection decisions on those values.

Our results also demonstrated that as ART triggers become more strict, the proportion of treatments adapted increases and the resulting distribution of metric values improves. Because the changes in target coverage metrics featured a skewed distribution, the curves representing the increase in proportion of treatments adapted were nonlinear. This suggests that the instances with the most compromised target coverage can be corrected using a relatively small number of online ART treatments. To improve the dosimetry further, however, will require adapting additional treatments at an increasingly rapid rate, leading to a diminishing return.

Numerous other studies have investigated the dosimetric effects of various online ART implementations. Precise, quantitative comparisons are challenging due to differences in technical details as well as the variety of metrics reported. However, general trends and dependences have continued to emerge. Li et al. compared non‐adaptive image‐guided radiotherapy, daily online ART, and a hybrid approach that combined a library of previously generated plans and some online plan reoptimization.^44^ In analyzing the CTV D99% for patients treated to the prostate plus seminal vesicles, they observed large decreases in target coverage due to anatomic changes (57.8%–104.1% of prescription dose), which were then recovered when using daily online ART (99.6%–105.1% of prescription dose). This was even the case when plans were not reoptimized for each treatment as per their hybrid method (98.7%–104.7% of prescription dose). This effect of compromised target coverage resolved by online ART is consistent with our findings, including when reoptimization was used only for a proportion of treatments.

More recently, Siciarz et al. analyzed eight different methods of implementing ART for patients with hypofractionated treatments to the prostate using a combination of online and offline methods.^45^ Their purpose was to discern a method that achieved adequate dose delivery without undue effort or demand for clinical resources. Included in their analysis was the change in several target coverage metrics also considered in this work. They observed that the CTV D99% and D95% both decreased on average about 4.5% compared to the original plan when not adapted. The values increased about 2% compared to the original plan when adapted on a daily basis. Similarly, the PTV D99% decreased approximately 11% and 2%, respectively, when never adapted or adapted daily. Maximum doses to the bladder and rectum did not demonstrate significant changes between the online ART plan and the original plan. These effects and magnitude of changes are also in‐line with our findings.

The treatment sites and techniques used in this work did not include nodal target volumes. Delivering dose to more extensive targets will likely influence the benefit online ART can provide for normal tissue sparing. Lutkenhaus et al. observed significant decreases in volume of the bowel receiving high doses for patients treated to the bladder with nodal volumes.^46^ Similarly, Kerkhof et al. found significant decreases in the volume of the bladder, rectum, and bowel receiving 45 Gy for patients treated to the cervix with nodal volumes.^47^ In the work presented here, the dosimetric effects of online ART on OAR metrics seemed largely random. Were more extensive target volumes used, OAR objectives might influence plan reoptimization more strongly. The OAR metrics that did demonstrate the most improvement with online ART were the bowel and rectum for patients treated with a sequential boost to the post‐operative prostate fossa. The fact that, in this case, the target is predominantly the post‐operative space may influence the anatomic variations and result in a greater opportunity for online ART to improve OAR dosimetry. These patients also exhibited the least amount of target coverage compromise, potentially allowing the adaptive plan to achieve greater normal tissue sparing. This illustrates the complex interactions between dose objectives that occur during plan reoptimization.

For managing anatomic variations, ART represents just one available tool and should be considered alongside others like margins and robust plan optimization. Sun et al. reviewed the anatomic variation observed for patients treated for cervical cancer and discuss the implications on target margins including with respect to ART.^48^ Anatomic variations can also be considered more explicitly during robust plan optimization.^49^ An early combination of ART and plan robustness is described by Birkner et al.,^50^ while Böck presents a framework for online robust ART including consideration of adaptation cost and frequency.^51^ Similarly, Liu et al. combine robust plan optimization and ART by using images acquired during the course of treatment to update the statistics that drive the robust optimization.^52^ This interaction between ART, margins, and robust optimization should continue to be explored as their optimal use is intertwined and dependent on many specific treatment planning and delivery considerations.

Interest in the determination and use of triggers has persisted throughout the history of ART. However, prior investigations have been dedicated to the initiation, timing, and patient selection for offline ART addressing longitudinal changes. Numerous authors have examined the use of target and OAR changes in geometry and dosimetry to trigger offline ART for sites like head‐and‐neck,^9^, ^10^, ^11^, ^12^, ^13^ soft tissue sarcoma,^14^ lung,^11^, ^15^, ^16^ cervix,^17^ and prostate.^11^ Triggers appropriate for determining the need for online ART during a particular treatment session, however, are in many ways functionally different from those appropriate for offline ART. As the unique benefit of online ART is to account for large, random, interfractional variations, the trigger information may not be available prior to the daily treatment, nor may it be relevant following the treatment. Lim et al. compared a midtreatment replanning strategy with a dosimetrically triggered strategy for offline ART patients treated to the cervix.^17^ While both strategies improved target coverage, only the former also improved dose to normal tissues. These observations may in part be attributable to the complex anatomic variations in patients treated for cervical cancer. As Lim et al. point out, their strategies are intended to account for tumor regression, a longitudinal effect. Yet the position and orientation of the cervical cancer target is also strongly influenced by daily variations in bladder filling that would best be addressed with online ART.

Furthermore, as Sonke et al. describe in their review of ART literature, “triggered adaptation has the disadvantage of being unpredictable.”^53^ This has a particularly strong consequence for online ART, where both the determination of the need to adapt and the adaptive intervention must occur in real‐time. As a result, with the current version of our system, the demand for resources required for ART are governed not by whether the adaptive plan is selected, but by whether an adaptive plan is even to be considered. This reinforces the value of meaningful, objective, and quantitative triggers that might facilitate decision making, empower more members of the clinical team, and be incorporated into expedient workflows.^54^ Considerations such as the trigger selection framework described here can help clinicians to resolve the levels at which adaptive interventions are likely to be of dosimetric consequence and to anticipate the required clinical resources.

To be used to this effect, online ART triggers that correspond with the best estimate of dose delivered to the patient should be determined. McCulloch et al. analyzed numerous potential offline triggers across several organs for head‐and‐neck cancer patients based on predictions of cumulative dose metric values.^55^ Online ART implementation will benefit from similar analyses that also consider the large, random, interfractional nature of the anatomic variations it is intended to address. Sibolt et al. describe their initial experience addressing such variations with implementation of CBCT‐based online ART for several sites in the pelvis.^18^ In their description, the authors found that the predominant reason for selecting to treat with the adaptive plan was to maintain target coverage rather than to improve OAR sparing. This is consistent with our observation that recovering compromised target coverage was a stronger signal than OAR sparing for dosimetric improvement from online ART, making target coverage metrics the basis for effective triggers.

Several considerations should be maintained for proper interpretation of the work presented here. First, our analysis only considered the dosimetric effects observed for individual treatment sessions. A more comprehensive estimate of the total dose delivered to a patient might be made by accumulating dose over multiple treatments. However, doing so also introduces additional uncertainty from deformable image registration and assumptions made during dose accumulation calculations. In addition, the relationship between accumulated dose and the conventional dose objectives normally used to evaluate the original treatment plan remain unknown. Considering each treatment individually can therefore be an effective way to assess how the original dose distribution is compromised within the scope and influence of the possible adaptive intervention.

A second limitation of this work is that each trigger was evaluated without consideration of other metrics. However, the conceptual framework described here can be extended readily to consider multiple metrics. Online ART could be implemented using multiple triggers combined with logical operators (e.g., adapt when dose coverage is compromised excessively *OR* when a high‐priority OAR dose limit is exceeded). In addition, the effect that a trigger based on one metric had on another metric was also not considered. When combining triggers and their effects across metrics in this way, the number of possible triggers is exceedingly large. The dimensionality and complexity of such considerations may be better approached through machine learning and artificial intelligence.

Third, the metrics investigated here were those corresponding with DVH‐type dosimetric objectives. To be evaluated, these require both a dose distribution and a structure contour, which, depending on the specific online ART workflow, may only be available following considerable time and effort during a treatment session. Nonetheless, the effects of various adaptive triggers could still be used at this point for clinical review or even be incorporated into automatic decision support tools. In addition, similar triggers could also be applied to estimates or predictions of the dose distribution and structure contours, potentially avoiding costs associated with generating them. Even in scenarios where the decision to adapt is derived from daily imaging, such methods may still implicitly incorporate approximate dose distributions or structure contours in which case triggers of the form presented here remain relevant. The decision to adapt can also be made based on geometric imaging values alone, although these metrics are less directly correlated with the dosimetric quality of various treatment plans.

Fourth, this work considered triggers that described the Scheduled Dose and Adapted Dose in relation to the Reference Dose. Also important are the absolute values of particular metrics as they relate to clinical dose objective tolerances. However, not only is it unclear as to how to properly interpret dose tolerances conventionally used for the Reference Dose now to be used for daily dose distributions like the Scheduled and Adapted Doses, but doing so increases the space of possible trigger parameters and values considerably. The decision to focus on dosimetric changes in relation to the Reference Dose was made for simplicity. Furthermore, the decision seems justified considering that the effects observed on target coverage metrics (which are frequently near the limits of dose objective values to avoid undue dose to normal tissues) changed considerably with the Scheduled Dose, such that the metric no longer achieved the clinically desired objective.

Fifth, this work represents our institution's experience with CBCT‐based daily online ART. Elements that are knowingly or unknowingly specific to our clinical operation and workflow may influence the observed changes in metric values which, in turn, determine the dosimetric and procedural effects. These institution‐specific elements include, but are not limited to, preferences in prescribing, contouring, planning, delivering, and managing treatment. In addition, the factors pertaining to the specific workflow of the existing CBCT‐guided ART system may influence the generalizability of our results. While the conceptual framework of adaptive trigger selection for online ART described here can be extended to future CBCT‐guided systems and even MR‐linac systems, our quantitative results can only be reported as to how they pertain to our specific experience.

Lastly, of course, the work would also benefit from increased patient numbers in order to improve statistics as well as to represent scenarios not yet observed in the patients that were available to be incorporated in this study. Increased patient numbers are particularly important for shorter treatment courses where the number of treatments per patient was limited.

Despite these limitations, this work provides previously unreported data and analysis with considerable implications for the effective use of online ART. This is the first analysis of this conceptual framework facilitating the study of trigger selection on data from clinical patients treated with CBCT‐based daily online ART as presented here across multiple pelvic treatment sites and techniques. This analysis addresses several clinical concerns such as the dosimetric effect of uncorrected interfractional variations, the ability of online ART to recover the original dosimetric values, and the magnitude of dosimetric improvement when delivering an adaptive plan instead of the original plan. In addition, this work demonstrates how observed dosimetric differences due to anatomic variations affect how ART trigger selection influences other consequences like the proportion of treatments adapted and the resulting distribution of metric values. In this work, it was shown that target coverage metrics were more sensitive to anatomic variations than OAR metrics, and that these trigger values resulted in a strongly nonlinear impact on the proportion of treatments adapted. These newly characterized effects and considerations will be critical to the successful implementation of online ART.

Our results can inform the implementation of online ART on both a patient‐specific and population level. We observed significant interpatient variations regarding the need for online adaptation to maintain adequate target coverage. While the utility of the framework described here does not require patient‐level specification, similar methods that incorporate patient‐specific information as it becomes available over the course of treatment may improve the precision in predicting the dosimetric and procedural effects of online ART. One simple strategy, included here for illustration, might be to treat a patient non‐adaptively for a period of time (e.g., 1 week), gathering datapoints that may better reveal the potential cost and benefit of online ART which would then be implemented as appropriate. This workflow would have the added benefit that multiple instances of contours could be included to generate patient‐specific targets.^56^, ^57^ The interpatient variations that were observed during this work remain incompletely explained or characterized. Identifying and integrating related factors into patient management as well as treatment planning and delivery may promote the effective and judicious use of online ART for individual patients. Considerable work in determining the optimal implementation of this CBCT‐guided ART system to maximize its impact at the patient and population level is currently ongoing at our institution.

On a larger scale, multicenter clinical trials could use analyses similar to those presented here to characterize population, sub‐population, and individual effects of online ART in greater detail by leveraging their larger sample size and strict trial protocols. This work suggests that, in addition to the specific parameters and values used for an online ART trigger, other treatment planning factors may strongly influence the consequence of using online ART. These factors include how contours are delineated, how they are to be used by the treatment planning and delivery systems, and what specific objectives and priorities are assigned to them during plan optimization. Clinical trial protocols should specify factors like these to ensure consistent implementation and valid scientific comparisons. Even outside of clinical trials, the impact of these factors implies that they should be reported by investigators when describing experimental methods and results.

## 5 CONCLUSION

Large, random, interfractional variations in patient anatomy compromise target coverage across pelvic treatment sites. Our clinical implementation of CBCT‐based daily online ART is able to recover the original target coverage. These effects were much larger than those observed for OAR metrics, suggesting that maintaining target coverage is the primary benefit of our use of daily online ART. This work observed a diminishing improvement in dosimetry when using increasingly strict triggers, and it also observed strong patient‐specific effects. Taken together, these suggest that developing methods able to discern the value of online ART of each individual patient is critical for the technology to be implemented in the most effective, yet resource‐conscious way. The concept of adaptive trigger selection—whether it is applied to a single trigger or multiple triggers, each based on the Scheduled and/or Adapted Doses—looks to be an effective framework with which to evaluate the critical balance between the dosimetric benefit and the clinical resource cost of various ART implementation strategies.

## AUTHOR CONTRIBUTIONS

Areas of expertise: TH‐ External beam‐ photons: adaptive therapy, TH‐ External beam‐ photons: Motion management‐ interfraction, TH‐ External beam‐ photons.

All the authors contributed to conception, data acquisition, data analysis, and writing of the manuscript.

## CONFLICT OF INTEREST STATEMENT

ADY reports a patent regarding an adaptive radiotherapy phantom.

## Data Availability

Research data are not shared.
